# Using evidence from observational healthcare data to inform a trial design

**DOI:** 10.1186/1745-6215-14-S1-O1

**Published:** 2013-11-29

**Authors:** Christian Holm Hansen, Gordon Murray, Michael Sharpe

**Affiliations:** 1 University of Edinburgh, Edinburgh, UK; 2 University of Oxford, Oxford, UK

## 

Clinical trials are expensive. Understanding where a trial is needed and what questions to address is therefore important. We ask if pilot work using observational healthcare data can be used to inform a trial.

Symptoms of significant psychological distress are common in cancer patients but are often left untreated. It is unknown to what extent such distress symptoms persist over time and require treatment. Should a clinical trial be devised to determine if patients would benefit from treatment? How many and what patients are likely to suffer from persistent distress and to require treatment? On what criteria should patients be included in a trial, and when should trial outcomes be evaluated?

We address these questions using repeated, psychological symptom data that were routinely collected from over 20,000 outpatients who attended selected oncology clinics in Scotland. However, the limitations to routinely collected observational healthcare data are many. We approach the analysis within a missing data context and develop a Bayesian model in WinBUGS to estimate the posterior predictive distribution for the incomplete longitudinal responses and covariates under both MAR and MNAR mechanisms and use this model to generate multiply imputed datasets for analysis in SAS. The findings are compared with those from a purpose-designed, prospective, clinical study in the same population.

Meaningful analysis of observational healthcare data requires subject-matter knowledge, good quality data and appropriate statistical methods. With flexible modelling techniques now more practicable, we believe pilot work with such data could help inform future trial designs.

